# Marked by stigma, inked by belief: tattooing in leprosy – a community perspective

**DOI:** 10.3205/dgkh000626

**Published:** 2026-02-17

**Authors:** Nimisha Kabra, Rajesh Sinha, Aditya Abhishek Jaiswal

**Affiliations:** 1 Department of Dermatology, Indira Gandhi Institute of Medical Sciences, Patna, Bihar, India; 2 Department of Radiodiagnosis, Indira Gandhi Institute of Medical Sciences, Patna, Bihar, India

## Abstract

**Introduction:**

Leprosy remains a public health challenge in India, not merely due to medical reasons but because of persistent stigma and deep-rooted cultural beliefs that influence health-seeking behaviour. This case report describes a 28-year-old woman with borderline tuberculoid leprosy who delayed seeking medical care due to cultural beliefs.

**Result:**

After an 8-month delay in seeking medical care, the patient presented with a hypopigmented, hypoesthetic patch over the right knee, along with thickening of the right common peroneal nerve. Notably, two sets of intersecting tattoo lines were observed over the lesion applied by a traditional healer in an attempt to contain the disease. Histopathology confirmed a diagnosis of borderline tuberculoid leprosy, and high-resolution ultrasonography corroborated peripheral nerve involvement. The patient was subsequently given multidrug therapy for multibacillary leprosy, adult regimen for 12 months and received detailed counselling on disease management.

**Discussion:**

The case highlights how cultural practices can hinder early diagnosis and effective management. Culturally sensitive health education is essential to combat stigma and improve leprosy control.

## Zusammenfassung

**Einleitung:**

Lepra ist in Indien nach wie vor eine Herausforderung für die öffentliche Gesundheit, und zwar nicht nur aus medizinischen Gründen, sondern auch wegen der anhaltenden Stigmatisierung und der tief verwurzelten kulturellen Überzeugungen, die das Verhalten bei der Suche nach medizinischer Versorgung beeinflussen. In diesem Fallbericht wird eine 28-jährige Frau mit tuberkuloider Borderline-Lepra beschrieben, die aufgrund kultureller Überzeugungen die Inanspruchnahme medizinischer Versorgung hinauszögerte.

**Ergebnis:**

Nachdem die Patienten acht Monate lang keine medizinische Versorgung in Anspruch genommen hatte, stellte sie sich mit einem deutlich abgegrenzten, hypopigmentierten, hypoästhetischen Fleck über dem rechten Knie vor, der mit einer Verdickung des rechten Nervus peroneus einherging. Über der Läsion befanden sich zwei sich kreuzende Tätowierlinien, die von einem traditionellen Heiler mit dem Versuch, die Krankheit einzudämmen, angebracht worden waren. Die Histopathologie bestätigte die Diagnose einer borderline tuberkuloiden Lepra, und die hochauflösende Ultraschalluntersuchung bestätigte die Beteiligung des peripheren Nervs. Die Patienten wurde daraufhin sechs Monate lang mit einer paucibazillären Multimedikationstherapie behandelt und erhielt eine ausführliche Beratung zum Krankheitsmanagement.

**Diskussion:**

Dieser Fall zeigt, wie kulturelle Praktiken eine frühzeitige Diagnose und eine wirksame Behandlung behindern können. Eine kultursensible Gesundheitserziehung ist unerlässlich, um die Stigmatisierung zu bekämpfen und die Leprakontrolle zu verbessern.

## Introduction

An unusual yet revealing case is described that not only draws attention to the persistence of leprosy in India but also sheds light on the powerful influence of cultural beliefs on health-seeking behaviour. Despite decades of public health initiatives and increased awareness, deeply rooted misconceptions continue to shape how certain communities perceive and respond to this disease. 

## Case description

**Figure 1:**
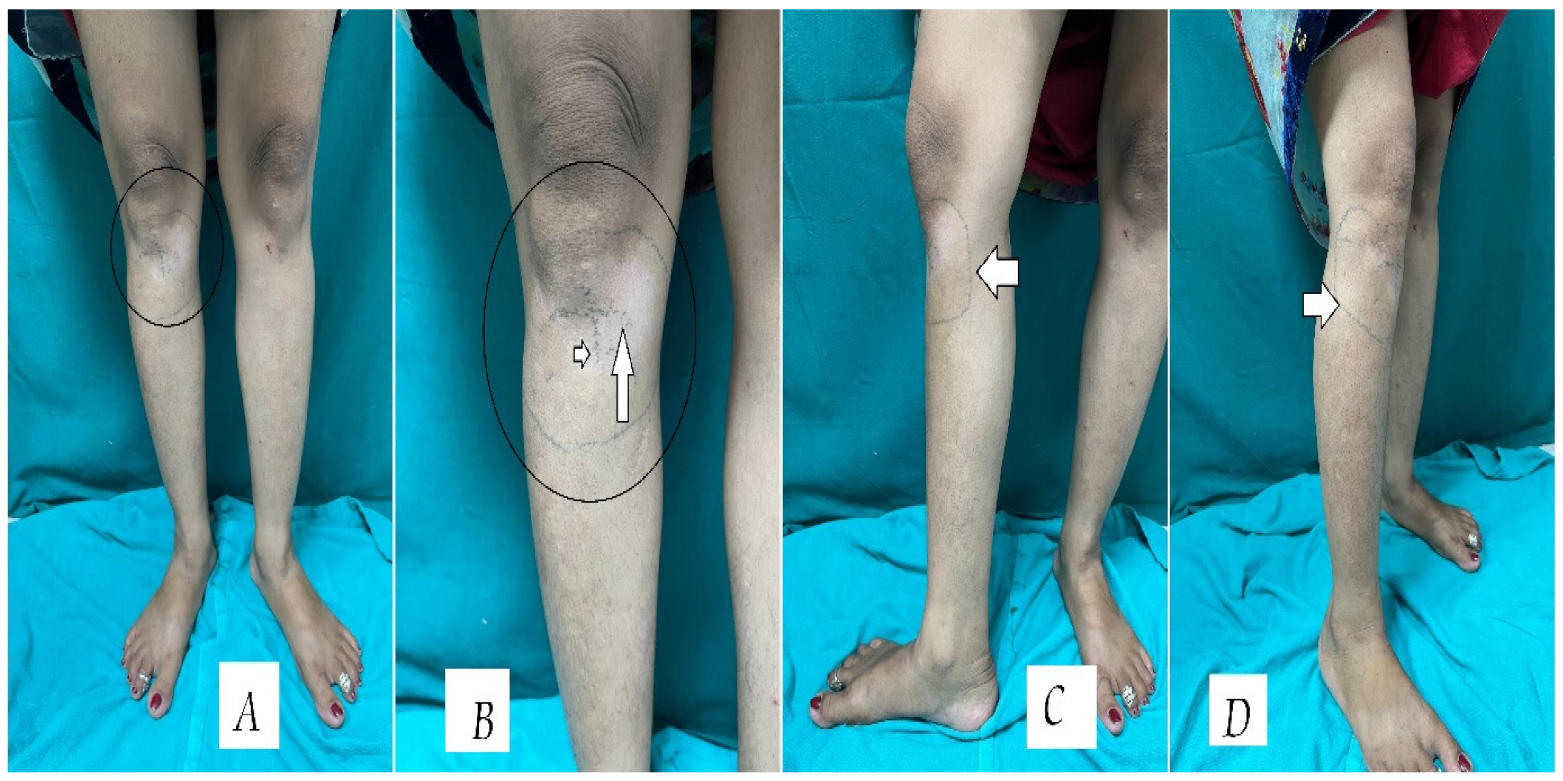
Unilateral hypoesthetic hypopigmented macular lesion over right lower knee extending over leg (A,B : encircled). B: Two sets of intersecting tattoo markings (white arrows) which was done by a traditional practitioner in her village as a form of “treatment” to contain the disease. C,D: A second set of dotted tattoo lines was added to encompass the new margins.

A 28-year-old female presented to our dermatology outpatient department with a hypopigmented, hypoesthetic patch below the right knee (Figure 1 (Fig. 1)) which had been present for 8 months. Initially the patch started as pea sized lesion which gradually progressed in size and was associated with loss of temperature and touch. On cutaneous examination, a hypopigmented patch of size 5x6 cm below knee extending to medial and lateral side of leg, which was associated with absent temperature sensation and decreased touch sensation. On nerve palpation thickening of the right common peroneal nerve was present.

**Figure 2:**
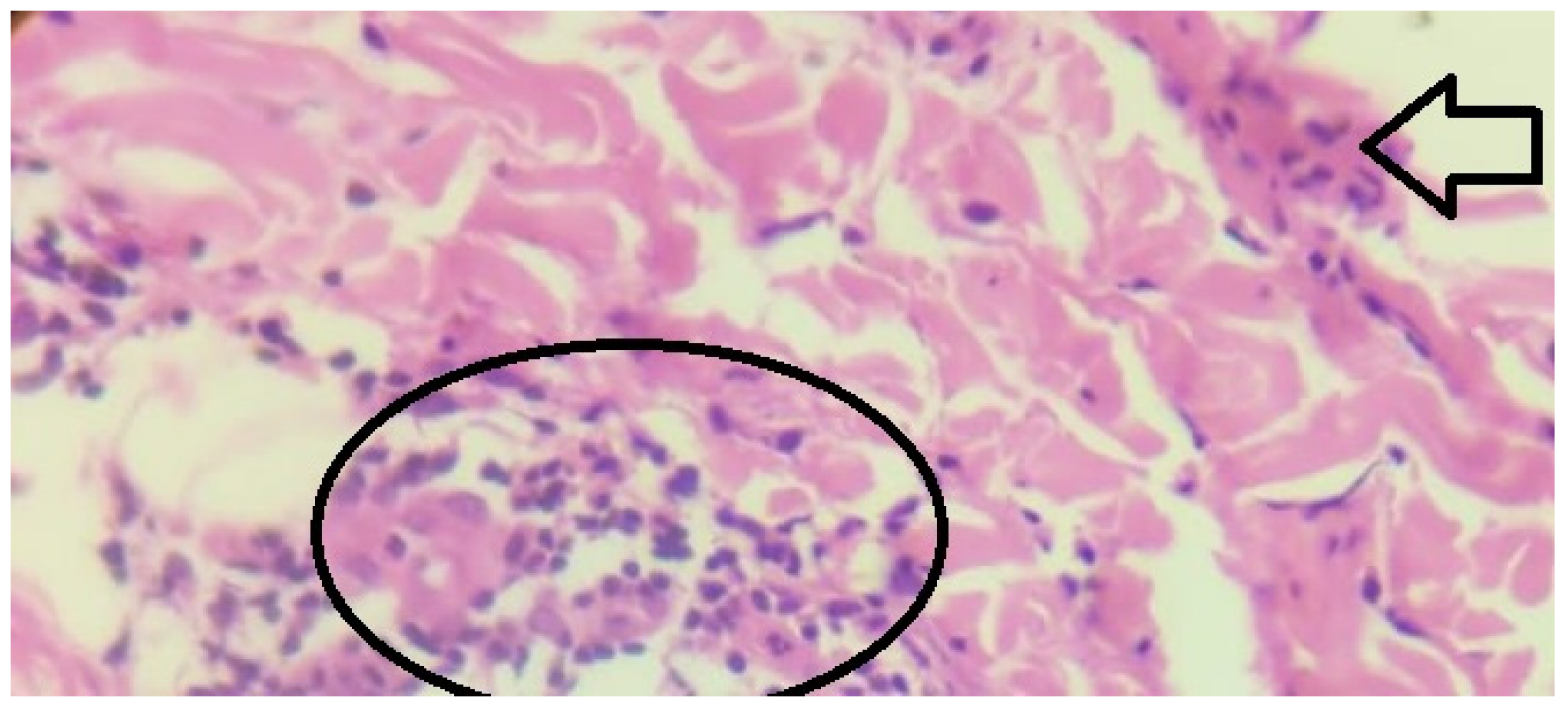
Hematoxylin- and eosin stained histopathological sample skin taken from the patch revealed epithelioid cells (black encircled area) with Langhans’ giant cells (white arrow, few present in encircled area).

Slit skin smear for AFB leprae was negative. A skin biopsy was done from the lesion and revealed epithelioid cells with Langhans’ giant cells (Figure 2 (Fig. 2)). 

**Figure 3:**
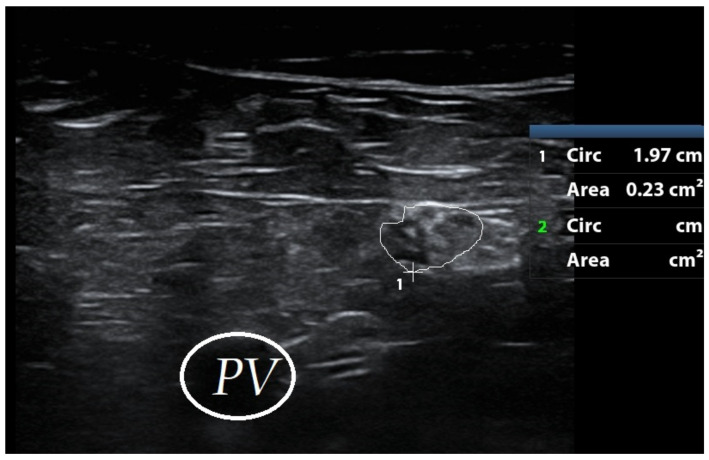
Increased cross-sectional area of right common peroneal nerve encircled with white line (Landmark: Popliteal vessels [PV], Normal cross sectional area – 6 mm^2^).

High resolution ultrasonography of the right common peroneal nerve revealed enlarged cross-sectional area (Figure 3 (Fig. 3)). 

Interestingly, two sets of intersecting tattoo markings were noted on the lesion. Upon further enquiry, the patient revealed that the initial tattoo, a set of two intersecting lines, was applied by a traditional practitioner in her village as a form of “treatment” to contain the disease within the boundaries of the lesion. However, as the patch enlarged despite the tattoo, a second set of dotted tattoo lines was added to encompass the new margins. This act was believed to "seal the infection" and prevent further spread (Figure 1 (Fig. 1)). The patient delayed seeking medical help during this time, relying entirely on these culturally influenced practices. The tattooing, done without medical supervision or hygiene, did not prevent progression and posed additional risks such as secondary infection and scarring. Patient was eventually diagnosed with borderline tuberculoid leprosy (Ridley-Jopling classification), and according to revised World Health Organisation (WHO) classification, she was classified under multibacillary leprosy due to the involvement of right common peroneal nerve irrespective of number of skin lesions, thus she was given multidrug therapy for multibacillary leprosy (MDT-MB adult) (capsule rifampicin 600 mg once monthly supervised dose, capsule clofazimine 300 mg once monthly supervised dose with 50 mg daily and tablet dapsone 100 mg daily) for 12 months and was counselled regarding the course of disease. 

## Discussion

This case exemplifies how traditional practices, often born of stigma and misinformation, can delay timely diagnosis and treatment. It serves as a reminder that addressing leprosy requires more than just medical intervention; it calls for a culturally sensitive, community-centered approach.

Leprosy, despite being a curable disease with free treatment available through national programs, continues to be surrounded by deep-rooted stigma and misinformation in many parts of India. This case illustrates how cultural beliefs can directly interfere with early diagnosis and effective treatment. The use of tattooing as a “barrier” to halt disease spread reflects a common misconception among some rural communities, where leprosy is still perceived as a supernatural or socially contaminating condition rather than a bacterial infection [1].

Such tattooing practices not only delay medical intervention but also expose individuals to the risk of secondary complications such as infection, scarring, or even nerve damage from repeated trauma to affected skin. The idea of marking lesions with ink or ash has been observed in some ethnographic studies on traditional healing practices in South Asia [2]. Furthermore, the act of concealing lesions or self-managing them through non-medical means often stems from the fear of social ostracization, loss of livelihood, or marital rejection which are the key elements of the leprosy-related stigma that persist despite public health awareness campaigns [3]. This stigma has been shown to contribute to significant diagnostic delay, higher disability rates, and mental health issues, such as depression or anxiety [4]. Public health responses must therefore go beyond medical provision and actively engage with cultural narratives. Training community health workers to identify and challenge such harmful beliefs, integrating culturally sensitive educational campaigns, and fostering trust in biomedical care are essential steps toward controlling leprosy transmission and reducing disability burden. Strengthening community-based awareness programs and integrating cultural understanding into public health strategies are vital for achieving sustained progress in leprosy elimination.

## Conclusion

This case highlights the profound impact of traditional beliefs on the course of a treatable disease like leprosy. The use of symbolic tattooing as a perceived remedy underscores the need to bridge cultural understanding with biomedical care. Misconceptions not only postpone appropriate therapy but also increase the risk of avoidable complications. Community education that respects local traditions while promoting scientific awareness is key to overcoming such barriers and achieving sustainable disease control.

## Notes

### Authors’ ORCIDs

- Kabra N: https://orcid.org/0009-0003-7361-9461
- Sinha R: https://orcid.org/0000-0003-0999-5772
- Abhishek Jaiswal A: https://orcid.org/0009-0009-1566-6415

### Ethical approval 

Written informed consent was obtained from the patient’s legal guardian for publication of this case report and any accompanying images. 

### Funding

None. 

### Competing interests

The authors declare that they have no competing interests.
